# Development and Production of Artificial Test Swarf to Examine Wear Behavior of Running Engine Components—Part 2: Experimentally Derived Designs

**DOI:** 10.3390/ma16186276

**Published:** 2023-09-19

**Authors:** Patrick Brag, Volker Piotter, Klaus Plewa, Alexander Klein, Mirko Herzfeldt, Sascha Umbach

**Affiliations:** 1 Department of Ultraclean Technology and Micromanufacturing, Fraunhofer Institute for Manufacturing Engineering and Automation IPA, Nobelstrasse 12, 70569 Stuttgart, Germany; 2 Institute for Applied Materials (IAM-WK), Karlsruhe Institute of Technology (KIT), Hermann-von Helmholtz-Platz 1, 76344 Eggenstein-Leopoldshafen, Germany; volker.piotter@kit.edu (V.P.); klaus.plewa@kit.edu (K.P.); a.klein@kit.edu (A.K.); 3 AuE Kassel GmbH, Heinrich-Hertz-Str. 52, 34123 Kassel, Germany; m.herzfeldt@aue-kassel.de; 4 Department of Machine Elements and Engineering Design (iaf), University of Kassel, Mönchebergstr. 7, 34125 Kassel, Germany; s.umbach@uni-kassel.de

**Keywords:** MicroPIM, particle, shape characterization, damage, tribology, bearing

## Abstract

In subtractive manufacturing processes, swarf, burrs or other residues are produced, which can impair the function of a tribological system (e.g., journal bearings). To prevent premature engine damage, cleanliness requirements are defined for production processes. Damaging particle tests are an experimental approach for validating these defined cleanliness requirements. This methodical approach is not yet widely used. For one, the test setup must be developed and proven for the respective application. For another, in order to carry out the tests in a systematic manner, defined test particles with properties similar to those of the contaminants encountered in reality are required. In the second part of the paper, the process chain for manufacturing artificial test swarf by micro powder injection molding (MicroPIM) is described. The size and shape of the swarf were derived from real swarf via several abstraction processes. Although certain design guidelines for MicroPIM parts could not be taken into account, the targeted manufacturing tolerances were achieved in most cases. During demolding, it became apparent that the higher ejection forces of the free-formed geometries must be taken more into account in the design of the mold. The experiments on the test setup also revealed that the artificial test swarf was unexpectedly brittle and was therefore ground up in the bearing gap without causing any substantial damage to the bearing. Thus, the artificial test swarf in its current sintered state is not a suitable substitute for micromilled swarf. However, MicroPIM could still be used to manufacture test particles in applications involving lower mechanical forces.

## 1. Introduction

The original motivation to carry out the project described in this paper was the commitment of automobile manufacturers to successively reduce fuel consumption and CO_2_ emissions [1]. In doing so, the limits of what is technically feasible are also being pushed in order to increase engine performance. Consequently, vehicle systems are subjected to higher loads or even overloaded when further requirements are added—this is what happened as a result of EU Directive 2000/53/EC [2]. Vehicles manufactured after 1 July 2003, may only contain small traces of lead. Up till then, the bearing shells in which the crankshaft runs were alloyed with lead. Lead was able to embed smaller particles, making the bearing shells relatively robust against contamination (so-called embedding behavior). Subject to the EU Directive 2000/53/EC, new coating systems or alloys had to be developed, which initially led to a deterioration in embedding behavior. Today, however, lead-free journal bearings perform better than those containing lead. At this point, a distinction is made between two types of contamination in terms of particle size and origin. Oil used to operate hydromechanical systems may contain particles in the submicrometer range. In this context, ISO 12669 describes a method for defining cleanliness requirements [3]. In analyses to assess wear on rolling bearings, reference is often made to the contamination in the oil [4,5]. When parts are machined, however, particles are generated that can be several hundred to a thousand micrometers in size [6]. In this context, ISO 16232 describes some approaches for specifying cleanliness, including the so-called damaging particle tests [7]. Wear resulting from insufficient particle cleanliness tends to occur in the mid to long term due to a large number of small particles. Arizona Test Dust is often used as a synthetic contaminant [8]. In 1990, the particle mixture Synform^®^ was developed, which is based on typical contaminants found in practice [9]. Today, Synform^®^ is no longer commercially available. Failures caused by slightly larger particles, on the other hand, occur much faster or immediately (within the warranty period). For damaging particle tests, however, larger particles are required, whereby the material, size and shape of individual particles must be considered as relevant parameters. Damaging particle tests have already been carried out in previous work [10]. For this, micromilled particles made of 42CroMo4 ranging from 500 µm to 2000 µm in length were used [11]. Similar to the evolution of crash tests, the question arose as to whether the scope of the tests could be reduced using Discrete Element Modeling (DEM) and Computational Fluid Dynamics (CFD) [12]. Through our own cleanliness analyses, we have learned that the uniformity of the particles is extremely low, which makes modeling more complicated. However, this aspect is not considered further here, as this project was concerned with the question of whether abstract particle shapes exhibit similar damage behavior to that observed with micromilled swarf (reference). To this end, a two-step approach was chosen. In the first step, five regularly shaped particle geometries were defined and tested, which were optimized for DEM and CFD simulation [13]. In this project—the second step—a particle shape was defined and tested, which was originally derived from real swarf encountered in industry. The expectation was that this shape would be more difficult to model for DEM-CFD, but in return, the damage behavior would be closer to that of the reference.

## 2. Design Development

**Figure 1:**
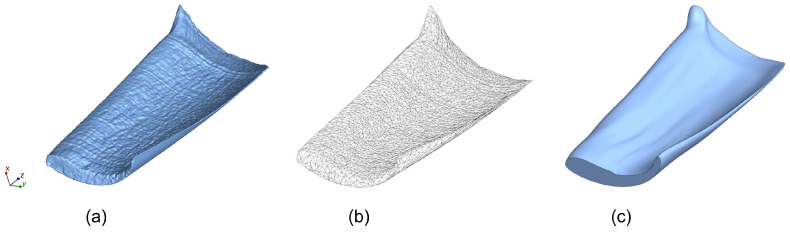
Reverse engineering of the particle shape: (**a**) CT scan of micromilled swarf. (**b**) Derived net model of the CT scan. (**c**) Smoothened volume of the net model.

To produce the artificial test swarf, micro powder injection molding (MicroPIM) was again used in the second step in order to maximize the reproducibility of the particle geometry. As a rule, swarf from industrial milling processes comes in an enormous variety of shapes and sizes. Therefore, selecting a representative swarf geometry initially proved to be quite difficult. However, this was facilitated by the fact that it could be assumed that the most serious damage potential would come from the largest swarf with a relatively flat shape. In addition, the results of a previous project [10] could be used, in which most of the tests were successfully carried out with micromilled particles 900 µm, 1100 µm and 1300 µm in length. The width of all particles was 400 µm, regardless of their length. The thickness was also constant, with the thickness gradient of a particle being max. 70 µm and min. 5 µm. The slightly helical shape and the kink about 150 µm along the length of the particles were due to the milling tool selected. [11] These relatively large dimensions also benefited the design of the MicroPIM mold or the micro-structured mold inserts. This was especially true for the diameters of the ejectors, which cannot be arbitrarily reduced given the current state of the art in precision mechanics. Ultimately, the decision was made to use micromilled swarf as a reference that was 1100 µm long, 400 µm wide and 70 µm thick, see Figure 1.

**Figure 2:**
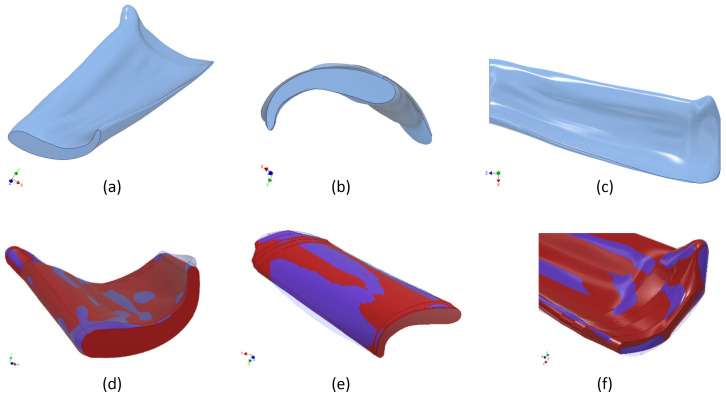
Design development based on the smoothened volume, resulting in the adjusted design of the artificial test swarf: (**a**–**c**) Perspective 1–3. (**d**–**f**) Altered sections, Perspective 1–3.

The next task was to derive a manufacturing-suitable design for both the micro test swarf and the molding tool while keeping as close to the original geometry as possible. With a view to future mass fabrication, the mold inserts should be produced using established precision engineering processes. To compensate for sintering shrinkage, all dimensions of the test swarf cavity were increased by a factor of 1.167 in line with a sintering shrinkage of 14.3%, which again was determined by the powder-binding ratio of 63 vol%. Although various micro manufacturing techniques have reached a remarkable performance, certain limits still have to be considered. As a consequence, several sections of the original swarf geometry needed to be significantly altered, see Figure 2.

**Figure 3:**
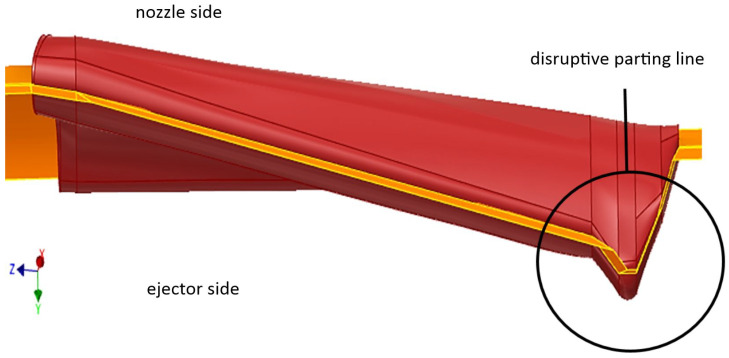
Parting line exhibiting critical disruptive section.

An important aspect was the correct positioning of the parting line, not only because a two-plate layout of the tool was still favored but also to avoid any demolding difficulties or even cutbacks. Additionally, the severely disruptive appearance of the parting line would have led to extremely short edge lengths of only 15 µm (in the mold insert cavities), which would have been impossible to fabricate by micro mechanical processes (Figure 3).

**Figure 4:**
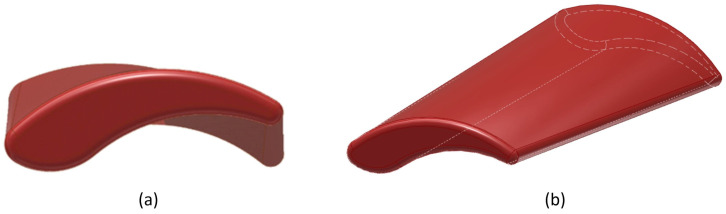
Final design of the artificial test swarf: (**a**) Perspective 1. (**b**) Perspective 2.

**Figure 5:**
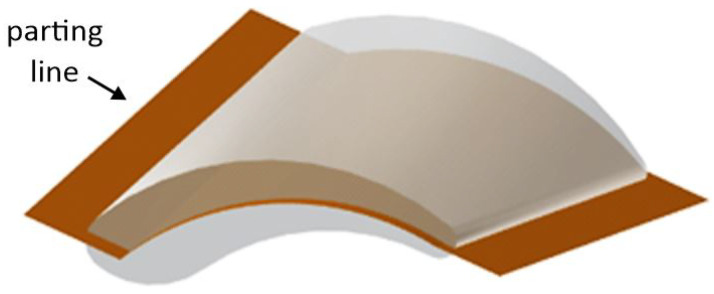
Final artificial test swarf design with straightened parting line.

Therefore, the swarf geometry needed to be further aligned, part thicknesses adjusted and the parting line replaced by a smoother one. The modified design, which could now be realized via precision die sinking, is shown in Figure 4 and Figure 5.

**Figure 6:**
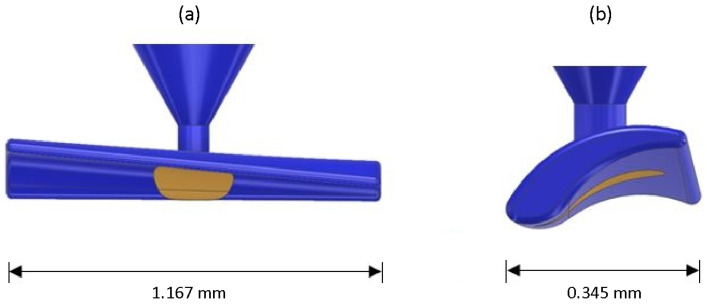
Schematic drawing of the artificial test swarf showing the gate and ejector position, the latter is shown in light brown: (**a**) Front view. (**b**) Lateral view.

The next task was to position the injection gate and ejector pin correctly. It soon became apparent that the best location was the middle of the swarf, see Figure 6. Further modifications concerned the implementation of a runner system with a gate diameter of 100 µm and an ejection pin whose front side formed part of the test particle cavity itself (so-called contour ejector).

**Figure 7:**
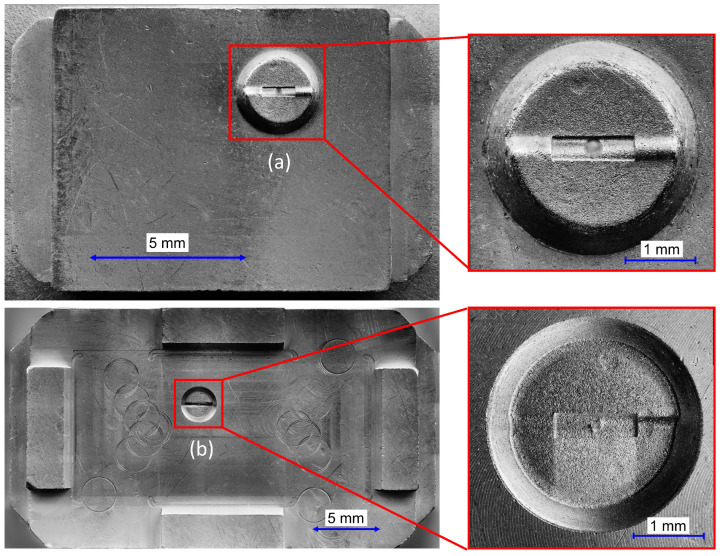
Mold inserts: (**a**) Ejection side with ejector hole. (**b**) Nozzle side with injection gate.

As a molding tool, an already existing one that only required modification was used. Figure 7 shows the mold inserts bearing the micro-structured sections.

## 3. Freeform Micro Powder Injection Molding Process

As large quantities of artificial test particles will be required, a typical fabrication process known as micro powder injection molding (MicroPIM) was chosen. A further advantage of this technique is the possibility to process the same steel materials as real swarf is made of. Detailed descriptions of the PIM manufacturing technology can be found in [14,15] and, with a certain focus on micro-related issues, in [16,17,18,19]. The real swarf design as characterized by CT represents, of course, a complex freeform geometry, i.e., it shows varying dimensions in all spatial directions. In order to replicate the design by injection molding, certain modifications had to be made, especially with respect to the present limitations governing the fabrication of mold inserts. Although the greatest challenge was the new freeform design, further modifications were necessary, especially in terms of feedstock development. For the abstracted test particles, low-alloyed 42CrMo4 (1.7225) heat-treatable steel widely used in combustion engines was chosen as a solid component. The powder was supplied by Sandvik Osprey Ltd. (Neath, UK) and had a D10 of 2.9 µm and a D90 of 5.7 µm as determined by laser interferometry. The BET surface was 0.25 m^2^/g. As a liquid component, the so-called GoMikro system, which has been well-proven at KIT for extremely small dimensions, was used. In this particular case, it consisted of paraffine wax, polyethylene, and stearic acid in a mixing ratio of 50/45/5 vol%. As already used in previous trials, the powder-binder ratio was set to 63 vol%. A laboratory measurement kneader was used for compounding. To shape the swarf by injection molding, a Wittmann Battenfeld Microsystem 50 machine well-known for its particular capability to produce micro-sized components, was deployed. Additionally, the so-called variothermal temperization method was used, i.e., prior to injection, the tool temperatures were heated up to keep the feedstock at a flowable level. Immediately after cavity filling, the tool was cooled down to achieve a strong green body and thus enable safe demolding. Injection molding parameters were derived from previous trials [13] with minor adaptations to the particular artificial swarf design being required. Finally, the parameters in (Table 1) were determined as the most suitable.

**Figure 8:**
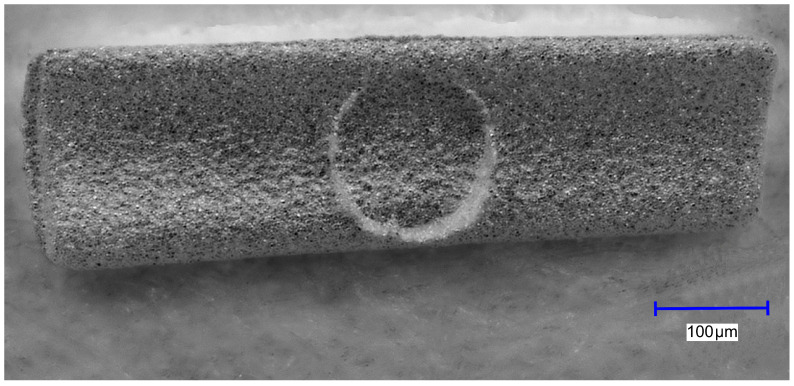
Typical green body produced by the micro injection molding process.

An example of such an injection-molded green body as seen under the light microscope is shown in Figure 8.

**Figure 9:**
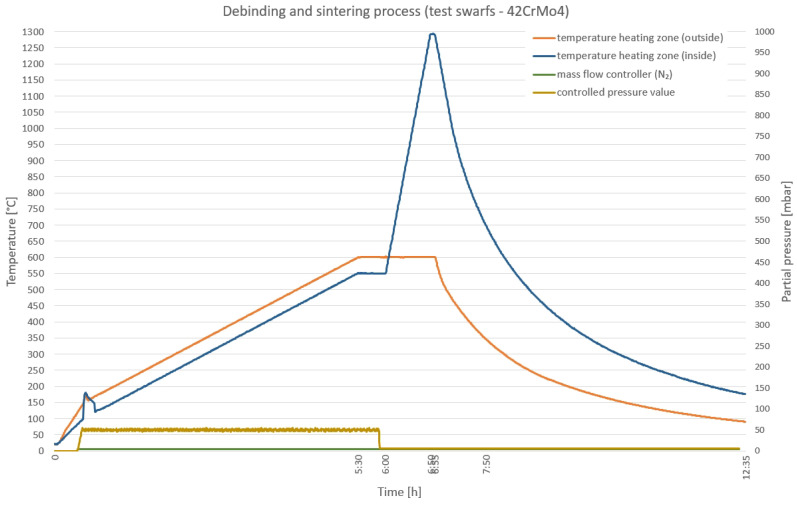
Diagram of the combined debinding and sintering heat treatment process. The upper curve shows the temperature in the sample chamber of the furnace. The maximum temperature was 1300 °C, held for only 5 min.

The green body has a length of approx. 1170 µm and a width of approx. 340 µm. Note the circular marking in the center caused by the ejector pin protruding a few micrometers into the injection molding tool. As usual, the GoMikro binder was eliminated in a two-step procedure: first by fluid dissolution in hexane to extract the wax component, followed by a thermal debinding step included in the heating ramp of the sintering procedure as depicted in Figure 9.

**Figure 10:**
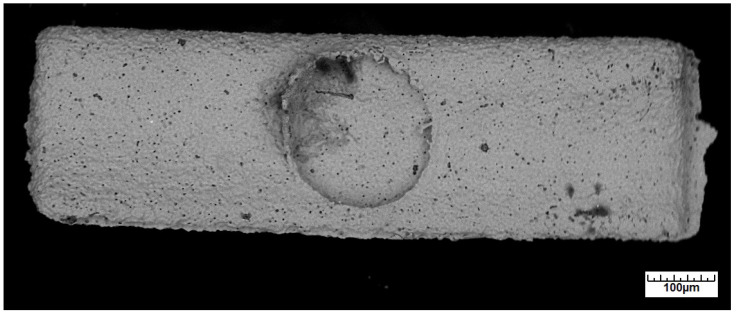
Finally sintered artificial test swarf.

In order to be as close to reality as possible, the sintering parameter had to be based on standard industrial data. The sintering recommendations published by commercial feedstock manufacturers are a valuable starting point for this. Typical and frequently adopted data sets can be found in [20]. It should be noted, however, that the dimensions of the test swarf are far below those of conventional industrial products, meaning that the sample volumes are heated through much more rapidly. To avoid excessive grain growth, the holding time at maximum sintering temperature was therefore significantly reduced. Together with thermal debinding to remove residues, this finally resulted in the temperature-time curve shown in Figure 9. An example of the finally sintered artificial test swarf is shown in Figure 10.

## 4. Results

The replication of real-world stress conditions due to the presence of manufacturing debris (natural particles) calls for artificial/synthetic test particles with equal characteristics in terms of:

- Strength/durability to exhibit equivalent damage behavior;
- Dimensional accuracy to achieve experimental repeatability.

In the first instance, the density of the finally sintered native swarf was measured to obtain an indication of strength capability. Obviously, measuring the density of micro-sized parts is not easy. The low amount of sample material and the tiny corners, for example, make this particularly challenging. As the classical Archimedes method reveals considerable uncertainties when testing such tiny samples, the density measurements were carried out using standard He-pycnometry. The measured value herewith was 7.02 g/cm^3^, corresponding to a theoretical density of 99.7% [21]. This result is a mean value obtained from several swarf particles, i.e., sample densities may vary considerably.

**Figure 11:**
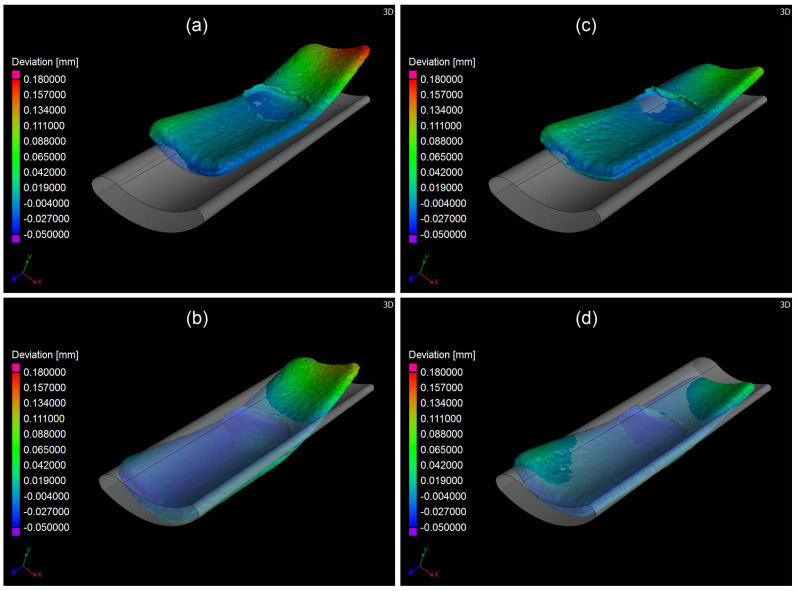
Nominal/actual comparison: (**a**) Best fitted artificial swarf no. 1; (**b**) Manual fitted artificial swarf no. 1; (**c**) Best fitted artificial swarf no. 22; (**d**) Manual fitted artificial swarf no. 22.

In the second instance, the dimensional deviations of the artificial test swarf were investigated by micro CT. The artificial test swarf were scanned with a resolution of 1.73 µm per voxel and aligned with the best-fit algorithm to the grey CAD model, offered by VGSTUDIO MAX 3.0.5 (Volume Graphics GmbH, 69115 Heidelberg, Germany), see Figure 11. The visual impression of the alignments resulted in a feedback test to achieve better manual alignments for swarf no. 1 and no. 22. Finally, the feedback test confirmed that the best-fit algorithm leads to the smallest deviations, despite the misleading visual impression.

**Figure 12:**
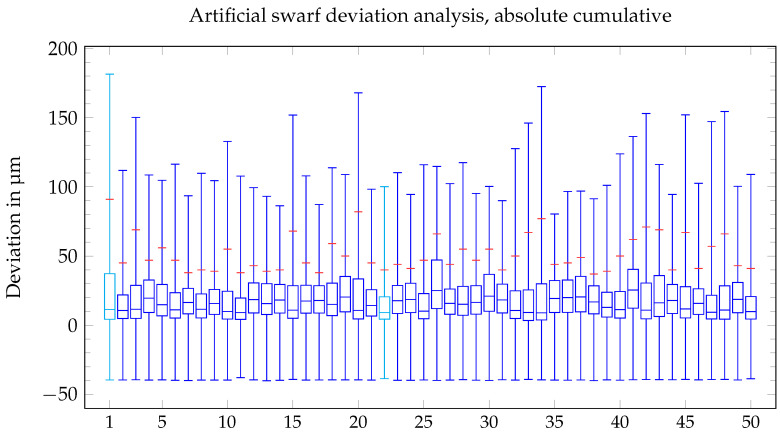
Box plot of the deviation analysis.

A previous survey showed good body accuracy of geometrically regular particle shapes produced by MicroPIM [6]. In contrast, the body accuracy of the native swarf did not meet expectations. Ejection from the tool caused a curvature in each of the analyzed artificial test swarf, resulting in an imperfect dimensional accuracy, see Figure 12. Dimensional accuracy was defined as 90% of all deviations shall be less or equal to ±10 µm, which corresponds to ±1.65 σ. The box plot reflects the cumulated 90% percentile of each artificial test swarf by a red line, which should have been at 10 µm (*y*-axis).

**Figure 13:**
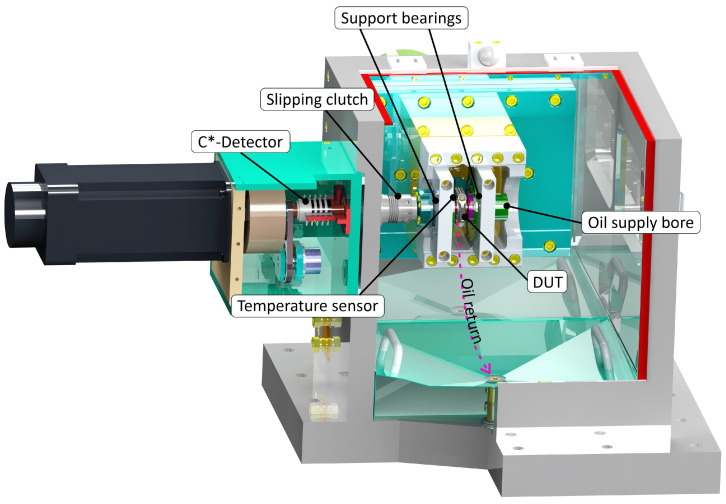
Journal bearing test rig.

In the third instance, the damage potential of the artificial test swarf was evaluated by means of tests on a journal-bearing test rig. The test setup consists of a triple-bearing shaft, two support bearings and a test bearing. A motorized load profile was applied to the test bearing via a hydraulic actuator synchronous to the speed. Oil was supplied to the test bearing via a bore hole in the shaft. The particles were fed to the test bearing via the same bore hole. Therefore, a particle injector was used (see Figure 13), which was developed in the BMWi joint project ‘Prüfpartikel’. This allows the particles to be introduced into the oil flow and conveyed by it to the interaction zone [13].

**Figure 14:**
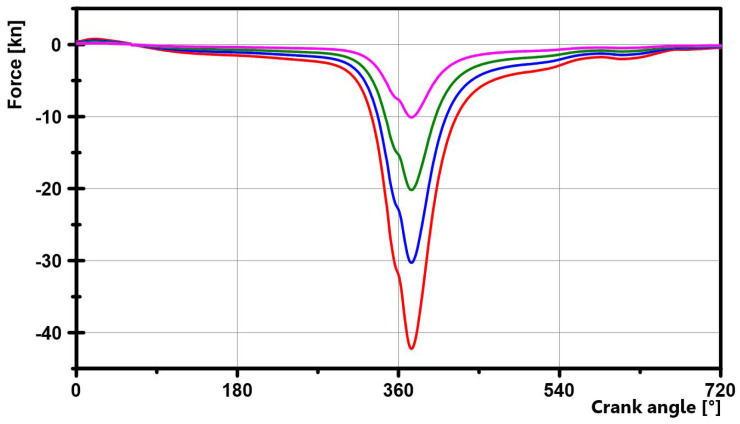
Run-in loads.

The test procedure was as follows:

1. Run-in with four load increments until the test load (pressure: 0 kN; tension: −42 kN) was reached. Duration: 30 min. See Figure 14.
2. Further operation with test load until constant operating conditions were reached, i.e., the temperature in the bearing and the C* value no longer changed.
3. Particles introduced via the oil supply inlet.
  (a) Test stopped after reaching maximum test duration (30 min). Here, damage levels varied between 1–5. The material wear/accumulation starting from Level 2 led to a deterioration in the performance of the bearings. Due to the so-called self-healing process, which is able to restore the hydrodynamic properties, a failure does not have to occur immediately. In practice, however, replacement would be highly advisable, as premature fatigue is to be expected.
  (b) Test aborted due to excessive bearing temperature (≥200 °C). The damage varied between 4–5. If the actual temperature rises above the typical bearing operating temperature, the bearing clearance decreases, and consequently, the proportion of dry friction increases, causing the actual temperature to rise further, and so on. This iterative process takes place within a few seconds, and particles do not necessarily have to be involved.
  (c) Test aborted due to excessive drive torque (≥28 Nm). The slip clutch opens within a few milliseconds when the threshold is exceeded. In these cases, the bearing shells were substantially damaged, which immediately led to bearing “seizure”. This corresponded to Level 5.
4. Connecting rod disassembled and bearing shells removed.
5. Cover and rod shells inspected for remaining swarf or damage.

The following damage levels were determined based on the tests carried out in the previous project with micromilled swarf [10]. The more severe damage tended to be caused by larger swarf.

**Figure 15:**
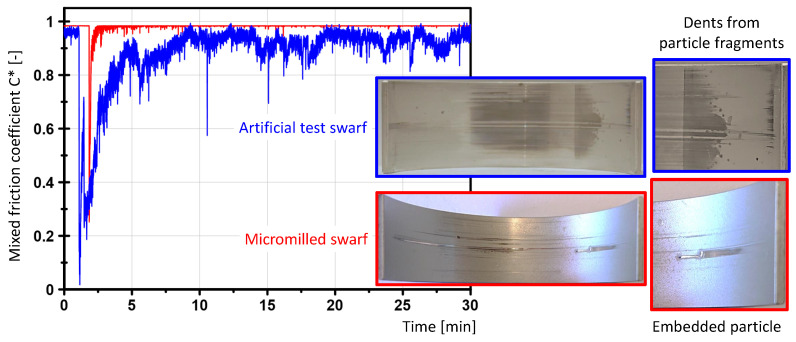
Mixed friction behavior of a test bearing after the interaction with an artificial test swarf.

The friction occurring in the journal bearing is a combination of hydrodynamic and dry friction. In a typical run-in phase, dry friction decreases in favor of mixed and liquid friction as wear progresses up to complete hydrodynamic friction, i.e., the normal operating point of a journal bearing. This condition changes immediately when the journal bearing is loaded with particles. The particles entering the gap between the shaft and bearing instantly cause dry friction, which may result in bearing seizure due to various damage mechanisms or alternatively disappear due to the so-called self-healing process of the journal bearing by renewed run-in. The measurement of the test bearings and the behavior during the test were used to assess the accumulated damage caused by the particles. Figure 15 shows an example of damage to a journal bearing caused by artificial test swarf after a test run.

**Figure 16:**
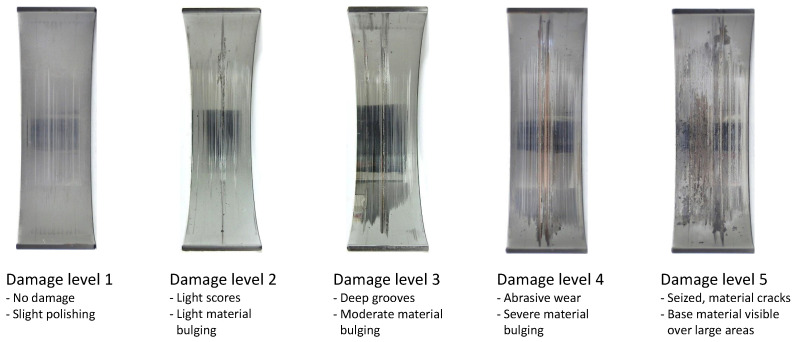
Damage levels caused by micromilled swarf.

Tests with the artificial test swarf showed a brittle behavior of the particles under the load in the interaction zone. The particles broke into many smaller fragments and left indentations in the bearing material. The system (shaft and bearing) needed several minutes to grind and flush out or embed the artificial test swarf, as shown by the decrease in the C* value. Only after about 10 min (Figure 16, blue curve) could the proportions of mixed friction be lowered to the previous level (before the addition of the swarf). The small fragments were not found in the bearing shells after Step 4 of the test procedure and were probably discharged with the oil return (cf. Figure 13). The example of the embedded micromilled swarf illustrates the completely different behavior of this type of swarf. In Figure 16 (red curve), mixed friction occurs only a few load changes after introducing the swarf and the system runs in the hydrodynamic range again within less than 1 min. On completion of the test, the swarf was found embedded in the bearing shell. In comparison, real swarf is harder and has sharper edges that tend to anchor or create grooves in the softer bearing material without breaking into pieces.

## 5. Conclusions and Outlook

Compared with the uniformly shaped test particles from Part 1 [13], the abstract test swarf could be successfully introduced into the bearing gap, as shown by the decrease in the mixed friction coefficient C*. However, the damage behavior differs from that exhibited by the micromilled particles of the same size. The damage pattern of the abstract test swarf is more comparable to that caused by a large number of smaller particles, which is known as pitting in rolling bearings [22], see Figure 15. In the reconstruction, the load from the engine must have crushed the abstract test swarf and washed it out of the interaction zone via the oil circuit to the reservoir. No embedded residues in the bearing shells were found after the tests. Only a small amount of the micromilled swarf remained in the bearing shells after the tests, but the deep grooves and furrows suggest that it was not ground up. The abstract test swarf produced have not yet reached the typical tolerances of the MicroPIM manufacturing process (up to ±0.3% of the nominal dimensions). This finding is quite understandable in view of the fact that basic design guidelines can only be taken into account to a limited extent in the free-formed geometries. The difficulties mentioned during demolding can very probably be eliminated by revising the mold concept. A further challenge is the need to optimize sintering parameters and to adapt thermal post-treatment steps such as hardening to the small dimensions of the swarf with their considerable dimensional differences. For damaging particle tests on (journal) bearings, where high mechanical forces are to be expected, micromilled swarf is still preferred given the current state of the art. Due to the higher manufacturing tolerances (up to ±100 µm), see [11], a large number of tests is required in order to achieve statistical significance. The use of DEM-CFD simulation to supplement the damaging particle tests still appears worthwhile from an economic point of view. For this, however, other manufacturing processes and particle shapes would have to be investigated with regard to manufacturing tolerances and damage behavior. It would be conceivable to produce the test particles by micro-embossing [23,24,25] and then to harden the ductile materials [26,27] in order to obtain the required strength. Again, abstract shapes would be feasible, which so far is the only approach for DEM-CFD simulation. In powder technology, simulations with non-spherical particles have been performed for several years [13], i.e., an abstraction as shown in Figure 1, Figure 2, Figure 3 and Figure 4 would not be necessary. In tribology, on the other hand, the particle shapes are composed of spherical basic shapes [28,29] since computer technology still reaches its limits due to the more complex elastic-plastic impact processes and fracture mechanics. Whether the approaches outlined will be pursued further in the automotive industry as originally planned is unclear as a result of the transformation toward electromobility. However, the results obtained here would be transferable to the power industry (wind turbines, gas turbines, etc.) or aviation industry, for example. In the broadest sense and expressed in general terms, the findings are applicable whenever parts manufactured using subtractive processes are moved in bearings.

## Figures and Tables

**Table 1:** Injection molding parameters applied for the artificial test swarf design [13].

Parameter	Value
Injection unit temperatures, zones 1–3	160/165/160 °C
Tool temperature, die side, injection	95 °C
Tool temperature, die side, demolding	25 °C
Tool temperatures, ejector side, injection	60 °C
Tool temperature, ejector side, demolding	20 °C
Injection velocity	125 mm/s
Injection pressure	1350 bar
Holding pressure	620 bar

## Data Availability

Not applicable.

## Abbreviations

The following abbreviations are used in this manuscript:

CAD	Computer Aided Design
CT	Computer tomography
BET	Brunauer, Emmet, Teller
MicroPIM	Micro Powder Injection Molding
PIM	Powder Injection Molding
